# Current Progress in Cancer Treatment Using Nanomaterials

**DOI:** 10.3389/fonc.2022.930125

**Published:** 2022-07-14

**Authors:** Ruirui Zhu, Fangyuan Zhang, Yudong Peng, Tian Xie, Yi Wang, Yin Lan

**Affiliations:** ^1^ Department of Cardiology, Union Hospital, Tongji Medical College, Huazhong University of Science and Technology, Wuhan, China; ^2^ Department of Dermatology, Union Hospital, Tongji Medical College, Huazhong University of Science and Technology, Wuhan, China; ^3^ Department of Cardiovascular Ultrasound, Zhongnan of Wuhan University, Wuhan University, Wuhan, China

**Keywords:** nanomaterial, cancer treatment, tumor microenvironment, drug delivery, chemotherapy, bioavailability, nanodrug

## Abstract

The pathological processes of cancer are complex. Current methods used for chemotherapy have various limitations, such as cytotoxicity, multi-drug resistance, stem-like cells growth, and lack of specificity. Several types of nanomaterials are used for cancer treatment. Nanomaterials 1–100 nm in size have special optical, magnetic, and electrical characteristics. Nanomaterials have been fabricated for cancer treatments to overcome cytotoxicity and low specificity, and improve drug capacity and bioavailability. Despite the increasing number of related studies, few nanodrugs have been approved for clinical use. To improve translation of these materials, studies of targeted drug delivery using nanocarriers are needed. Cytotoxicity, enhanced permeability and retention effects, and the protective role of the protein corona remain to be addressed. This mini-review summarizes new nanomaterials manufactured in studies and in clinical use, analyses current barriers preventing their translation to clinical use, and describes the effective application of nanomaterials in cancer treatment.

## Introduction

Cancer is a common, complex, and heterogeneous disease. As the population ages, cancer is becoming a leading cause of morbidity and mortality worldwide, with approximately 9.5 million cancer-related deaths annually (1). Therefore, studies aimed at developing treatments for cancer are urgently needed. Surgery, chemotherapy, and radiotherapy are the three main treatments for cancer but often lead to unsatisfactory outcomes and side effects (2, 3). With increasing advances in oncology research, more effective therapies have become available to overcome these limitations, such as immunotherapy, gene therapy, photothermal therapy (PTT), photodynamic therapy (PDT), chemodynamic therapies (CDT), sonodynamic therapy (SDT) and nanomaterial-based chemotherapy (4–9). Among them, nanomaterial-based chemotherapy is a promising therapy for cancer because of its low toxicity, high specificity, and excellent bioavailability (10).

Medical nanotechnology is one of the most promising frontiers in cancer. Typical nanomaterials are 1–100 nm in size and exhibit a high surface to volume ratio, unique fluorescence properties, enhanced permeability, and outstanding biocompatibility (11, 12). These properties offer several advantages in cancer treatment. The high surface to volume ratio facilitates the assembly of biomolecules, which can improve the specificity of chemical drugs, thereby increasing the efficacy of targeted therapy and reducing its toxicity (10).

PDT and PTT are promising approaches for improving the effects of cancer treatment; both methods are related to optical interference. The materials used in PDT and PTT have obtained wide interest in recent years. The temperature of the targeted region can be elevated by the photothermal conversion efficiency of PTT to promote cancer cell death. In contrast, in PDT, photosensitizers and certain wavelengths of light are utilized to produce singlet oxygen, leading to the death of cancer cells (13). Because of their excellent fluorescence properties, various nanomaterials are utilized in PDT and PTT (14). In tumor site, toxic ROS was generated by endogenous H2O2 and therefore cancer cells were killed in the absence of external O2 (15). This is one of the unique advantages of CDT. So far, CDT nanodrugs with mitochondria-targeting displayed outstanding spatial specificity and anti-tumor effect (9).

Nanoparticles are a better choice for cancer treatment compared to microparticles because nanoparticles are more biodegradable than microparticles (16). Nanoparticles are not small enough to penetrate into normal blood vessels because they have a dense extracellular matrix. As tumor grows, lymphatic drainage was inhibited by immature vasculatures generated by tumor-induced angiogenesis (17). The suppressed lymphatic drainage makes it possible for nanoparticles to penetrate targeted cells. This phenomenon is known as “enhanced permeability and retention effect” (EPR) and the passive targeting of nanoparticles is largely dependent on EPR effect (18).

The superparamagnetic behavior of nanomaterials facilitates the diagnosis and treatment of cancer. For example, superparamagnetic iron oxide nanoparticles offer unique advantages. They are small, display high targeting specificity, and show a powerful ability to evade the immune system, thereby exhibiting potential for cancer treatment (19).

## Advances in Nanotechnology for Targeted Delivery

**Figure 1:**
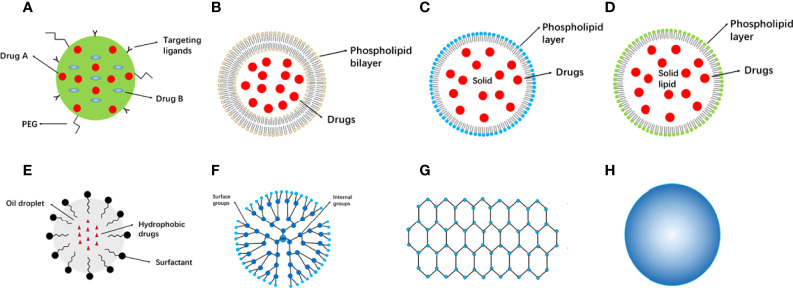
Categories of nanomaterials used in cancer therapy. **(A)** Nanoparticles. **(B)** Liposomes. **(C)** Solid lipid nanoparticles. **(D)** Nanostructured lipid carriers. **(E)** Nanoemulsions. **(F)** Dendrimers. **(G)** Graphene. **(H)** Metallic nanoparticles. PEG, poly (ethylene glycol).

Cancer treatment based on nanomaterials shows advantages over using free drugs, particularly for targeted delivery. Compared to free drugs, targeted delivery exhibits reduced toxicity, decreased degradation, increased half-life, and enhanced capacity (20, 21). Recent advances have been made in nanomaterial-based targeted drug delivery systems, including in active or passive targeting. Active targeting is achieved using antibodies or small molecule-conjugated nanoparticles, whereas passive targeting occurs through enhanced permeability and retention effects. Active targeting displays great potential and acted as an alternative strategy to passive targeting and the ability of tumor localization in active targeting was improved by increased efficiency and retention (22). Compared with traditional chemical therapies, nanomaterial-based drugs display increased specificity, improved bioavailability, lower cytotoxicity, better loading capacity, and a longer half-life. To date, many nanomaterials for cancer treatment have been developed based on remarkable advances in nanoscience, technology, and cancer pathology. However, few nanomaterial-based drugs have been intensively studied and utilized in clinical practice. Nanomaterials can be broadly classified into several categories (**Figure 1**).

## Nanomaterials in Cancer Therapy

Several well-studied nanoparticles are listed in **Supplementary Table 1** (23–28). Chemical drugs can be delivered and sustainably released to target sites by Polymeric nanoparticles (PNPs) (10 to 1000 nm) (29). Nanoparticle components have evolved over the past few decades; they were initially manufactured from non-biodegradable polymers, including polymethyl methacrylate and polyacrylates (30, 31). Because they cause chronic inflammation and toxicity, one challenge to using these types of PNPs is their timely removal. To overcome this difficulty, biodegradable polymers such as polylactic acid, poly (lactic-co-glycolic acid), and poly (amino acids) have been fabricated (32), which exhibit excellent advantages depending on their structures and properties. PNPs protect drugs from degradation, improve loading ability, and increase stability (33). However, metal nanomaterials are not considered for cancer treatment because renal and brain toxicity and denaturation of enzymes may be caused by excessive heavy metal element in-take (34, 35).

**Figure 2:**
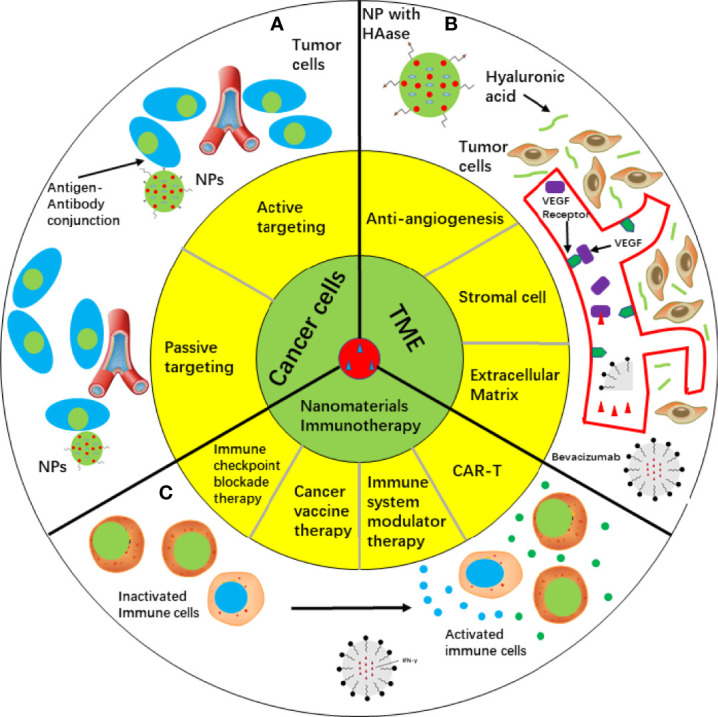
Cancer therapy approaches depending on nanomaterials. **(A)** Targeting cancer cells by passive targeting or active targeting. **(B)** Targeting TME, such as anti-angiogenesis, stromal cell and extracellular matrix. Bevacizumab was loaded in liposome and conjugated with VEGF to suppress angiogenesis. HAase was fabricated onto NP surface and penetration ability of NP was increased. c IFN-γ as an immune regulator delivered by liposomes activated immune cells in cancer immunotherapy. HAase, hyaluronidase; IFN-γ, Interferon gamma; NP, nanoparticle; TME, tumor microenvironment; VEGF, vascular endothelial growth factor.

Drug delivery plays an important role in cancer therapy, and non-conjugated polymers with intrinsic luminescence show its special advantage in drug delivery (36). Drug delivery are including passive and active targeting (**Figure 2A**). A crucial issue is the size of the drug. It is difficult for drugs to infiltrate the dense extracellular matrix. However, tumor induced angiogenesis promoted generation of numerous immature vasculatures and inhibited lymphatic drainage (37). Because these immature vasculatures are “leaky”, nanoparticles are small enough to penetrate the target sites. It is convenient to attach targeting peptides to the surface of PNPs because of excellent surface to volume ratio. Polysorbates surfactant effect can promote solubilization and fluidization. Coating polymers with polysorbates can improve bioavailability, thereby promoting the interaction of PNPs with endothelial cell membranes of the blood-brain barrier and facilitating endocytosis (38, 39). Compared with conventional chemical drugs, PNPS can deliver anticancer drugs to target sites and react to ultrasound. Theragnostics is a novel strategy that includes both diagnosis and therapy, in which fluorescent PNPs play a crucial role. In recent years, fluorescent PNPs with complex structures have been used as theragnostic materials. Fluorescent PNPs are typically composed of biocompatible biopolymers, fluorescent proteins, and organic dyes (40). Moreover, the anticancer effect of nanomedicine is strengthened by hydrophobic interactions or π–π bonds according to fluorescence assays (41). Radionuclides, such as I-125, can be stored in the stable core by electrophilic aromatic substitution of nanoparticles (42, 43). In addition, a self-assembling protein nanoparticle (11 nm) was developed and showed stability and biocompatibility in vivo, revealing its potential for drug delivery in cancer therapy (44). Ultrasound-sensitive PNPs are effective for cancer diagnosis and therapy. Side effects were reduced by using ultrasound in nanoparticle fabrication to overcome “obstacles” (such as endothelial blood vessels, tissue endothelium, and blood-brain barrier) associated with the traversing ability of cancer treatments, thus enhancing drug delivery (45–47). Ultrasound displays a thermal effect and can be utilized to trigger the controlled release of chemical drugs (48). However, some degraded PNPs are toxic; therefore, their fabrication and properties are still to be improved (49).

## Monoclonal Antibody Nanoparticles

Because of their anticancer effect and specific targeting ability, monoclonal antibodies (mAbs) are widely used in targeted treatment. More recently, mAbs have been utilized in anticancer nanoplatforms and as frontlines in the fight against cancer. Cytotoxic drugs are conjugated with mAbs to strengthen the therapeutic efficacy of anticancer drugs, known as antibody-drug conjugates. According to the specific antigens expressed in cancer cells, less toxicity and higher specificity can be achieved (50). Different antibody-drug conjugate systems display enhanced therapeutic efficacy in breast cancer (26, 51). Based on these effects of antibody-drug conjugates, trastuzumab nanoparticles are promising and widely studied nanoplatforms for cancer treatment (52–54).

## Lipid-Based Nanomaterials

Liposomes, solid lipid nanoparticles (SLNs), and nanostructured lipid carriers are three main categories of lipid-based nanomaterials. Liposomes (20 nm to >1 μm) were the first microcosmic phospholipid bilayer nanosystem (55). Both hydrophilic and hydrophobic drugs can be delivered depending on the liposome structure (56). Drugs are shielded from degradation by the central cavity of liposomes (57). Liposomes may be phagocytized by the mononuclear phagocyte system, known as human guards; therefore, liposome membranes should be modified to prolong their half-life (58). This can be achieved through polyethylene glycol conjugation. For example, PEG-liposomes carrying doxorubicin (DOX) were developed and applied to treat Kaposi sarcoma (59). Liposomes are widely applied in co-delivery and controlled release and have been combined with chemical drugs. How to load drugs and control release must be considered when designing liposome nanocarriers. Drug efficacy is affected by bioavailability in cancer chemotherapy, and DOX liposomes have a lower bioavailability than free DOX, suggesting that bioavailability should be improved during liposome design (60). A new PEGylated liposome carrying cobimetinib and ncl-240 displayed an enhanced cytotoxic effect through synergistic effects, leading to higher efficacy (61). Moreover, liposomes loaded with floxuridine and irinotecan exhibited better effects on advanced solid tumors, whereas a new liposome containing multilayer siRNA molecules and that co-delivered DOX displayed better DOX efficacy, decreasing the tumor mass in breast cancer (62, 63). Notably, specific liposomes can release drugs depending on the pH value, as cancerous regions are more acidic compared to healthy tissues (64). pH-sensitive cationic liposomes were prepared using a pH-sensitive material. The release of sorafenib was increased at pH 6.5 (65). In summary, liposomes exhibit low immunogenicity, low cytotoxicity, and high biodegradability (66). However, the disadvantages of liposomes include their rapid removal by the mononuclear phagocyte system, low stability, and obstacles in membrane transfer. Therefore, the application of liposomes remains limited.

SLNs have been acted as alternative carriers to liposomes. Because of the rigid confinement of the scale, SLNs (1–100 nm) are known as “zero-dimensional” nanomaterials compared with other larger nanomaterials. SLNs contain solid materials, in contrast to liposomes. Examples of these materials include solid lipids, emulsifiers, and water. PEGylated lipids, triglycerides, and fatty acids are utilized in SLNs (20). The outer layer and delivery function of SLNs are similar but show some differences from conventional liposomes. Some SLNs have a micelle-like structure rather than a contiguous bilayer, with drugs packaged in the core (67). Compared with liposomes, SLNs show higher stability and longer release. However, because of their crystalline structure, SLNs exhibit some limitations, such as inherently low incorporation rates and an unpredictable gelation tendency (68). Nanostructured lipid carriers (NLCs) have been fabricated to overcome the drawbacks of SLNs and termed as the second lifetime of lipid nanoparticles. Compared with SLNs, NLCs exhibit a higher loading capacity and show a less inclination of gelation (69). NLCs have received considerable attention in recent years because many drugs used in cancer therapy are lipophilic and can be administered through various routes (oral, parenteral, inhalational, and ocular) (70). NLCs are manufactured as systems that carry both liquid and solid lipids. Over the past two decades, the stability and loading capacity of NLCs have evolved.

## Nanoemulsions

Nanoemulsions (NEs) are colloidal nanoparticles with an aqueous phase, emulsifying agents, and oil (71). NEs are widely utilized drug nanocarriers. They have a lipophilic surface and solid spheres; three typical NE types are water-in-oil NE systems, oil-in-water NE systems, and bi-continuous NE (71). Compared with most lipid-based nanomaterials, NEs show the advantages of optical clarity, excellent biodegradability, and improved molecule release profiles (72). In contrast to conventional delivery vehicles, nanoemulsions are superior in efficacy and stability, and a serial of routes they can use to administer (73, 74). Intranasal route was used to carry small interfering RNA to the brain by nanoemulsions. In glioblastoma, CD73 small interfering RNA was successfully carried to the brain for gene silencing in a cationic nanoemulsion and show its great potential in drug delivery (75). Co-delivery using NEs can be performed to improve the effects and bioavailability of drugs. A previous study showed that an NE carrier containing paclitaxel (PTX) and Spirulina polysaccharides displayed improved anticancer effects by regulating the TLR4/NF-κB signaling pathways (76). An NE system composed of rapamycin and bevacizumab was developed. Increased inhibition of tumor migration and angiogenesis and improved cytotoxicity against cancer cells were observed in advanced melanoma (77) (**Figure 2B**).

NEs can also be used in immune therapies. A modified NE loaded with interferon-γ inhibited the viability of MCF-7 breast cancer cells and enhanced the activity of phagocytes. These results indicate the potential of these NEs for cancer therapy (78) (**Figure 2C**). A new NE carried baicalein and paclitaxel was produced enhanced oxidative stress and decreased glutathione levels in MCF-7/Tax cells, providing a suitable approach for increasing the sensitivity of cells to paclitaxel (74, 79). Compared to conventional paclitaxel production, baicalein-paclitaxel NE displayed higher anticancer efficacy in vivo (74, 79). These studies indicate that applying elaborately fabricated NEs is useful for managing multi-drug resistance.

NEs are transitioning to clinical application but require many improvements, despite their potential advantages. A high temperature and pressure are typically needed to fabricate NEs, for which few materials are suitable. It is difficult to commercially manufacture NEs. Moreover, compared to other conventional production methods, homogenizers and microfluidizers are often used to prepare NEs, making the process more energy-efficient. Further studies of NE metabolism are needed to evaluate the safety of NEs in clinical applications (72).

## Dendrimers

Dendrimers (1–15 nm) exhibit an architecture with highly branched surfaces (80, 81). Dendrimer molecules are composed of three parts: a central core, branches, and an exterior surface. A few dendrimers have been investigated for cancer therapy, including polyamidoamine (PAMAM), polypropylenimine, 5-aminolevulinic acid, and triethanolamine (82).

Depending on their unique structure, dendrimers exert specific functions, such as adjustable branches, outstanding solubility, and excellent bioavailability. Cationic dendrimers can form complexes with nucleic acids because of their positively charged surfaces. Hence, dendrimers can be utilized as efficacious nucleic acid nanocarriers. PAMAM and polypropylenimine have been extensively investigated. A PAMAM dendrimer composed of two parts was developed for simultaneously monitoring cancer cells and managing multi-drug resistance through fluorescence imaging. The first part was a carbon dot/DOX complex and the other part was composed of five PAMAM dendrimers with cyclic arginine-glycine-aspartic acid and d-α-tocopheryl polyethylene glycol 1000 succinate (TPGS). These two parts were combined via electrostatic interactions to obtain a PAMAM dendrimer. The carbon dots produced fluorescence, and arginine-glycine-aspartic acid ligands targeting αvβ3 integrin receptors induced targeting specificity; TPGS showed a remarkable ability to inhibit cancer cells (83).

Dendrimers are also useful for co-delivery. Tumor necrosis factor-related apoptosis-inducing ligand (TRAIL) is an important factor in the apoptotic pathway. In addition, colon cancer is typically treated using DOX. TRAIL and DOX plasmids were carried in a dendrimer nanocarrier, which showed enhanced anticancer effects compared to modified carriers containing the TRAIL or DOX plasmids alone (84). Depending on the dendrimer, a PAMAN nanocarrier was prepared and used to treat liver cancer. PAMAN dendrimers without decoration exhibit inefficient cellular internalization, low transfection efficiency, and unstable encapsulation; however, the competitive characteristics of nanomaterials exhibit many advantages in combination therapy (85, 86).

## Graphene

Because of its important mechanical and electronic characteristics, graphene has been widely studied in cancer therapy (87). Based on their composition, structure, and characteristics, single-layer and multi-layer graphene, graphene oxide (GO), and reduced graphene oxide have been defined (88). Graphene shows unique mechanical and electrochemical advantages. Optical transmittance, high density, and hydrophobicity are outstanding properties (89, 90). In addition, high drug-carrying ability and thermal conductivity are important properties in cancer theragnostic (91, 92). However, the poor solubility of graphene causes toxicity and prevents its manufacture (93). Hence, more bioavailable graphene-based nanomaterials are needed. A classical GO molecule is composed of functional oxygen groups, carbonyl groups, and epoxy groups (94). GO and the reduced derivative of GO display improved biological effects compared to graphene. Powerful hydrophilicity contributes to dispersions in aqueous solvents, avoiding aggregation induced by van der Waals and hydrophobic interactions (95). Additionally, nanosheets are a versatile platform on which hydrophilic functional groups can be combined with various materials, offering many advantages in targeted therapy and cancer diagnosis (96).

Direct immunogenicity is exhibited in graphene compared to in other nanomaterials, and the extent of the immunostimulatory capability can be modulated by changing the lateral size (97). Macrophages and dendritic cells can be activated by graphene, revealing their potential for cancer therapy. TNF-α was remarkably increased by 6-armed GO in RAW264.7 macrophages without altering IL-1β and IL-6 levels (98). Graphene can also suppress some cancer cells. Treatment of human osteosarcoma cells and normal osteoblasts with GO displayed significant cytotoxicity against osteosarcoma, and the apoptosis rate of osteosarcoma cells was remarkably higher than that of hFOB1.19 normal osteoblast cells (99). Cancer stem cells (CSCs) are cancer cells with self-renewability and high tumorigenic potency; these cells are associated with multi-drug resistance and interact with the TME (100). Hence, damage to CSCs is vital for preventing malignancy. GO can distinctively target CSCs and suppress several pivotal signaling pathways, such as Notch and STAT signaling. Moreover, GO can induce CSC differentiation (101). This effect was named as differentiation-based nanotherapy. However, the anti-CSC phenomenon are still to be evaluated.

## Nanomaterials for Cancer Immunotherapy

Nanomaterials exhibit great potential in promoting the efficacy of cancer immunotherapy. Cancer immunotherapy include two aspects: cancer vaccines and TME modulation. Cancer vaccines are developed to present cancer antigen to DCs and promote robust effector T-cell response, while TME modulation aims to expand the ability of cytotoxic T cells for killing cancer cells (102, 103). Moreover, nanomaterials preloaded with targeting ligands can be taken up by specific cells (104). Interestingly, a recent research reported that a D-enantiomeric supermolecule nanoparticle was fabricated and its p53-dependent antiproliferative activity was exhibited and therefore antitumor immunity was enhanced (105). Immunotherapy will be benefited from delivery of tumor antigens by nanomaterials and the immune response can be also modulated by nanomaterials because of their specific characteristics (106, 107). Of note, the stimulator of interferon genes pathway was activated by the PC7A nanoparticles and therefore contribute a robust response in anti-tumor immunotherapy (108).

## Bioinformatics in Cancer Research

Recently, bioinformatics gained lots of interest in the medicine and especially in the field of cancer research. The best cancer therapy depends largely on the right identification of the origin of cancers. With the developments of bioinformatics, machine learning methods have been used to predict the origin of cancer, depending on the mRNA expression, or miRNA expression. An interesting publication showed that tissue specific miRNA and DNA methylation markers have been used to detect the origin of cancer and classifiers based on DNA methylation are more powerful than miRNA-based classifiers (109). Moreover, multimodal deep learning was successfully used in the identification of choroidal neovascularization activity (110). The above classifiers and relevant biomarkers (such as mRNA) will promote the specific therapy of cancer in the future.

## Discussion

Nanomaterials display different properties, such as composition, structure, and immunogenicity. Cancer treatment based on these nanomaterials have been widely studied. Diverse modification can be obtained on nanomaterials and anti-tumor drugs can be preloaded into nanocarriers. Both the characteristics of the nanoplatform and agents are of vital importance. The efficacy, targeting ability, and biocompatibility are based on the modification of the nanomaterials. Drugs, peptide and small molecules can be carried by nanomaterials in targeted delivery and non-targeted delivery. In this mini-review, we focused on the properties of common nanomaterials and evolution of their applications in cancer treatment. Of note, we summarized the categories of nanomaterials used in cancer therapy and diverse cancer therapy approaches depending on nanomaterials. Nanoplatforms can also be designed to target the TME environment. With further studies of the pathophysiology of cancer genesis and multi-drug resistance germination, additional nanomaterial-based therapies will be explored. Although extensive research has been conducted, few nanomaterial-based therapies are currently in clinical use, and thus further studies are needed to address this issue. Overall, the development of nanobiotechnology will lead to progress in clinical translation. Nanomaterial-based drugs can lead to beneficial effects in patients with cancer.

## Author Contributions

RZ, FZ, and YP contributed equally to this work. RZ, FZ, and YP wrote the manuscript. YL, YW and TX edited the manuscript. RZ and FZ finished the figures. All authors contributed to the article and approved the submitted version.

## Conflict of Interest

The authors declare that the research was conducted in the absence of any commercial or financial relationships that could be construed as a potential conflict of interest.

The reviewer WZ declared a shared affiliation with the author YW to the handling editor at the time of review.

## Publisher’s Note

All claims expressed in this article are solely those of the authors and do not necessarily represent those of their affiliated organizations, or those of the publisher, the editors and the reviewers. Any product that may be evaluated in this article, or claim that may be made by its manufacturer, is not guaranteed or endorsed by the publisher.
