# Supplementary material for: Current Progress in Cancer Treatment Using Nanomaterials
Source: Front Oncol. 2022 Jul 14;12:930125. doi: 10.3389/fonc.2022.930125 (PMC9330335; doi:10.3389/fonc.2022.930125)
Supplement: Supplementary file 1 [file Table_1.docx]

**Supplementary**Table 1 Summary of NPs in cancer therapy

| Modification | Load | Treatment | Target cancer model | Outcome | | Reference | | | |
| --- | --- | --- | --- | --- | --- | --- | --- | --- | --- |
| PLGA NP | PTX | Chemotherapy | Human prostate cancer lines PC3 | Drug delivery efficiency was highly improved | | | [17] |  |  |
| Tmab modified NP | Docetaxel | Targeted therapy, chemotherapy | Human HER2-postive BT474 cells and HER2-negative MCF7 cells | Increased cytotoxicity in HER2-positive BT474 cells but not in HER2-negative MCF7 cells | | | [18] |  |  |
| Tmab modified NP | Paclitaxel | Targeted therapy, chemotherapy | Human HER2-postive breast cancer cell lines: BT474, SK-BR-3;HER2 negative cell line: MDA-MB-231 | | Better treatment efficacy and lower cytotoxicity | | [19] |  |  |
| PEG transferrin modified NP | Nucleic acids | Nucleic-acid-based therapy | Human prostate cancer lines PC3 Chronic myelogenous leukemia cells K562 | | Showed higher efficiency over untargeted particles | | [20] |  |  |
| PLGA NP | Alantolactone Erlotinib | Targeted therapy | Human pancreatic cancer cell lines PANC-1 and Patu8988T | | The synthesized NP induced significant cancer cell apoptosis and displayed anticancer effect | | [21] | |  |
| Exosome | Doxorubicin | Chemotherapy | Human breast cancer cells MDA-MB-231; Mouse ovarian cancer cells; Breast and ovarian cancer mouse models | | Cytotoxicity of doxorubicin was increased and drug accumulation in mouse heart was avoided | | [22] | |  |
